# Laparoscopic surgery for colorectal cancer in a patient with intestinal malrotation: a case report

**DOI:** 10.3389/fonc.2025.1579104

**Published:** 2025-11-19

**Authors:** Dipesh Kumar Yadav, Min Li, Jiahao Xu, Sisi Lin, Dandan Bao, Hanzhang Huang, Zhangwei Yang, Yiren Hu

**Affiliations:** Department of General Surgery, Wenzhou People’s Hospital, The Third Clinical Institute Affiliated to Wenzhou Medical University, Wenzhou, China

**Keywords:** intestinal malrotation, colorectal cancer, laparoscopic right hemicolectomy, D2 lymph node dissection, preoperative imaging

## Abstract

Intestinal malrotation (IM), a rare congenital anomaly, poses significant surgical challenges when coexisting with colorectal cancer—an association reported in fewer than 60 cases globally, predominantly in Japan. This study presents the first documented case in China of ascending colon cancer with nonrotation IM, successfully managed via laparoscopic right hemicolectomy with D2 lymphadenectomy. A 79-year-old female presented with abdominal pain, weight loss, and melena. Colonoscopy revealed an ascending colon mass, confirmed as adenocarcinoma. Preoperative contrast-enhanced CT demonstrated left-sided colonic malposition, an “M”-shaped colon, and anomalous ileocolic vasculature originating from the left of the superior mesenteric artery. A multidisciplinary team employed meticulous preoperative planning, utilizing multiplanar CT reconstruction to navigate anatomical complexity. Laparoscopic surgery involved adhesiolysis, division of Ladd’s bands, and D2 dissection, achieving R0 resection (pT3N0M0) with 50 mL blood loss and no intraoperative complications. The patient recovered uneventfully, discharged on postoperative day 9, with no recurrence at 18-month follow-up. This case underscores the feasibility of minimally invasive techniques in IM-associated malignancies when guided by embryological insight and advanced imaging. Key technical strategies included adaptive port placement, stepwise anatomical correction, and vascular tracing to ensure oncological adequacy. The successful integration of laparoscopic principles with anomaly-specific modifications highlights a paradigm for managing such rare presentations. Our findings emphasize that IM’s anatomical complexities need not preclude laparoscopic benefits, advocating for global case-sharing to refine standardized protocols and expand minimally invasive options in complex surgical oncology.

## Introduction

Intestinal malrotation (IM) is a congenital anomaly caused by incomplete rotation and fixation of the midgut during embryonic development, typically occurring between the 4th and 12th weeks of gestation (1, 2). While IM is most frequently diagnosed in neonates (incidence: 1 in 500–6,000 live births), its presentation in adults is rare and often asymptomatic (3). Symptomatic cases may manifest as chronic abdominal pain, nausea, vomiting, or acute complications such as bowel obstruction or volvulus (4). Notably, the coexistence of IM with colorectal cancer is exceptionally rare, with fewer than 60 cases reported globally—predominantly in Japan (5, 6). To our knowledge, this represents the first documented case from China. This unique clinical scenario poses significant diagnostic and surgical challenges due to the abnormal anatomy complicating preoperative planning and intraoperative execution.

In recent years, laparoscopic surgery has become the standard of care for colorectal cancer, offering advantages such as reduced postoperative pain, faster recovery, and shorter hospital stays compared to open surgery (7). However, its application in IM-associated cases remains technically demanding and underreported. Among the limited global cases, only 10 were managed purely laparoscopically due to aberrant anatomical features—including atypical vascular patterns, mesenteric adhesions, and altered bowel positioning—that hinder oncologically adequate lymph node dissections (2, 5). These challenges have historically favored open surgical approaches in such complex presentations.

The scarcity of published data highlights the need for detailed documentation of technical strategies, safety profiles, and outcomes in these rare cases. By sharing our experience, we aim to contribute to the growing body of evidence supporting the feasibility and safety of laparoscopic surgery in managing colorectal cancer with IM.

We present a case of ascending colon cancer with IM that was successfully managed via laparoscopic right hemicolectomy with D2 lymph node dissection. This case presentation was approved by the ethics committee of Wenzhou People’s Hospital. It was prepared in accordance with the CARE case report guidelines (8).

This report emphasizes the critical role of preoperative imaging, meticulous intraoperative anatomical mapping, and strict adherence to oncological principles in addressing the challenges posed by IM. By detailing the technical nuances and outcomes of this approach, we provide a valuable reference for surgeons encountering similar challenges and demonstrate the potential of minimally invasive techniques in this complex clinical scenario.

## Case presentation

A 79-year-old female presented with a one-week history of persistent, dull pain in the left upper abdomen, which was non-radiating and exacerbated after meals. She reported no prior abdominal surgeries but had experienced occasional episodes of melena over the past year, which she attributed to hemorrhoids. At presentation, she was hemodynamically stable, with normal vital signs and no signs of acute distress. The patient also described a one-month history of progressive anorexia, early satiety, and unintentional weight loss of approximately 5 kg. Physical examination revealed mild tenderness in the left upper quadrant without palpable masses or organomegaly.

**Figure 1:**
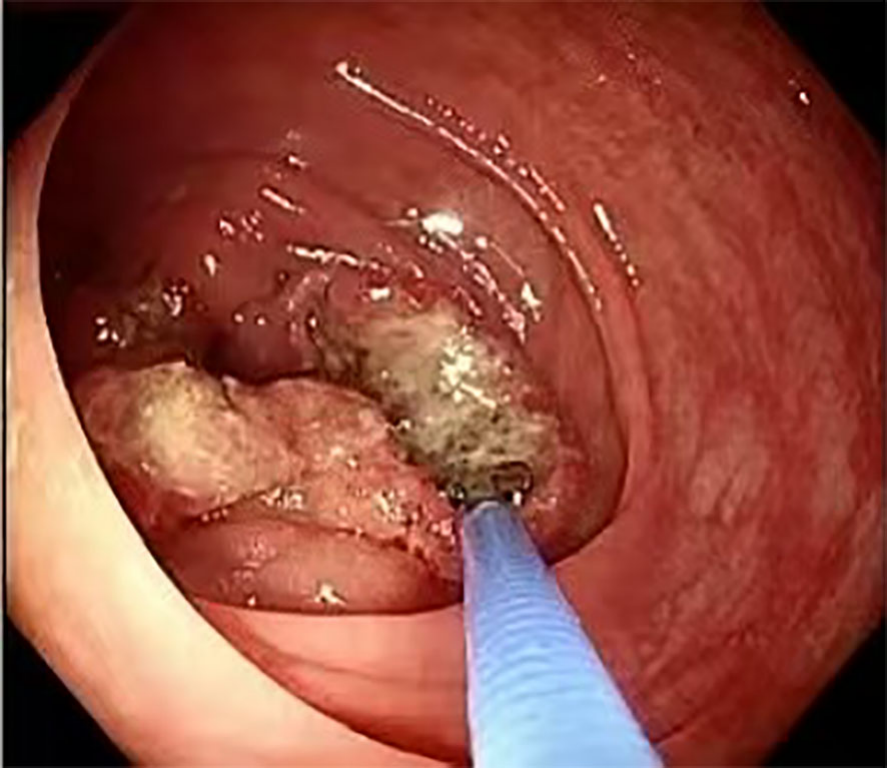
Colonoscopy showing a proliferative, ulcerative lesion in the ascending colon.

Colonoscopy was performed to investigate her symptoms, revealing a proliferative, ulcerative lesion in the ascending colon, approximately 90 cm from the anal verge. The lesion had irregular margins and a longitudinal diameter exceeding 5.0 cm, raising suspicion for malignancy (**Figure 1**). Histopathological examination of the biopsy showed moderately differentiated adenocarcinoma with no evidence of mucinous or signet-ring features.

Laboratory investigations revealed mild anemia (hemoglobin: 10.2 g/dL) and elevated carbohydrate antigen 19-9 (CA19-9) levels at 115.8 U/mL (normal: <37 U/mL), while carcinoembryonic antigen (CEA) levels were within normal limits at 2.6 ng/mL (normal: <5 ng/mL). Liver and renal function tests were unremarkable.

**Figure 2:**
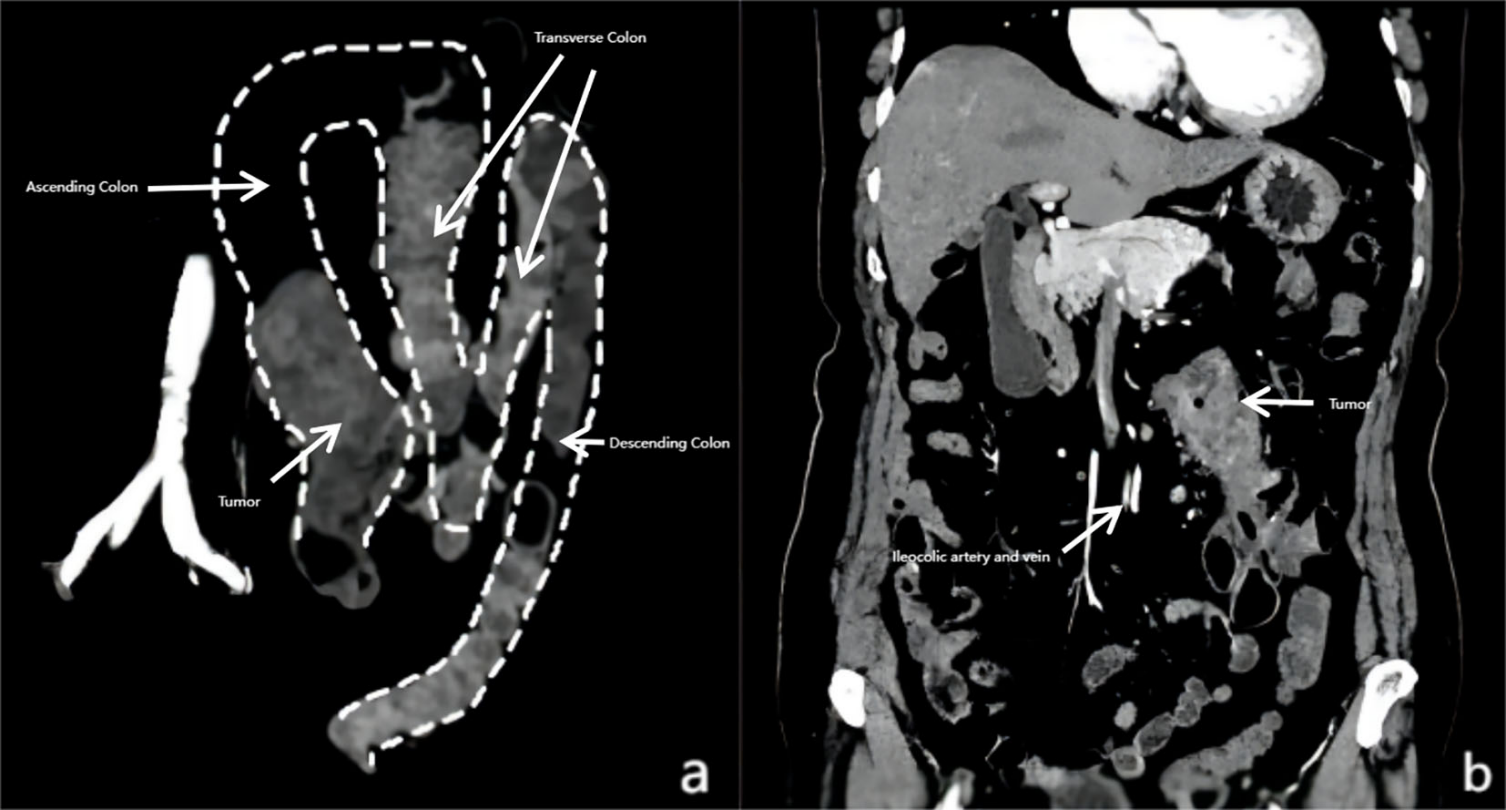
Computed tomography (CT) scan showing colorectal cancer with intestinal malrotation. **(a)** CT scan showing the cecum and right colon located on the left side of the aorta, forming an “M” shape due to dense adhesions among the transverse, ascending, and descending colon. **(b)** The ileocolic artery and vein originating from the left side of the superior mesenteric artery (SMA), which were positioned to the left of the abdominal aorta.

Preoperative contrast-enhanced computed tomography (CT) of the abdomen and pelvis was performed for staging and surgical planning. The scan identified intestinal malrotation. Sagittal reconstruction of the CT scan demonstrated the entire colon located on the left side of the abdomen, forming an “M”-shaped configuration. This configuration was due to dense adhesions among the ascending, transverse, and descending colon. Additionally, the cecum was located in the left side (**Figure 2a**). The scan also revealed a 5.5 cm mass in the proximal ascending colon, consistent with malignancy, and several suspicious enlarged regional lymph nodes, the largest measuring 1.2 cm in short-axis diameter. Notably, the ileocolic artery originated from the left side of the superior mesenteric artery (SMA), with the accompanying ileocolic vein also located on the left side of the SMA (**Figure 2b**). No distant metastases were identified in the liver, lungs, or other abdominal organs.

Although the presence of enlarged lymph nodes would typically necessitate D3 lymphadenectomy for optimal oncologic control (9), the multidisciplinary team recommended D2 lymphadenectomy due to prohibitive risks arising from the convergence of complex anatomical factors—including IM, dense adhesions, aberrant left-sided ileocolic vascular anatomy, and tumor location—combined with the patient’s advanced age and comorbidities (well-controlled hypertension and type 2 diabetes). This decision prioritized surgical safety, as contemporary evidence demonstrates that D3 dissections increase mortality and elevate complication rates compared to D2 dissections, particularly vascular injuries and organ damage in elderly patients (10).

Given these compounded technical and patient-specific risks, the patient was extensively counseled regarding anticipated intraoperative challenges, including a high probability of conversion to open surgery. Informed consent was subsequently obtained from the patient’s relatives.

## Surgical procedure

**Figure 3:**
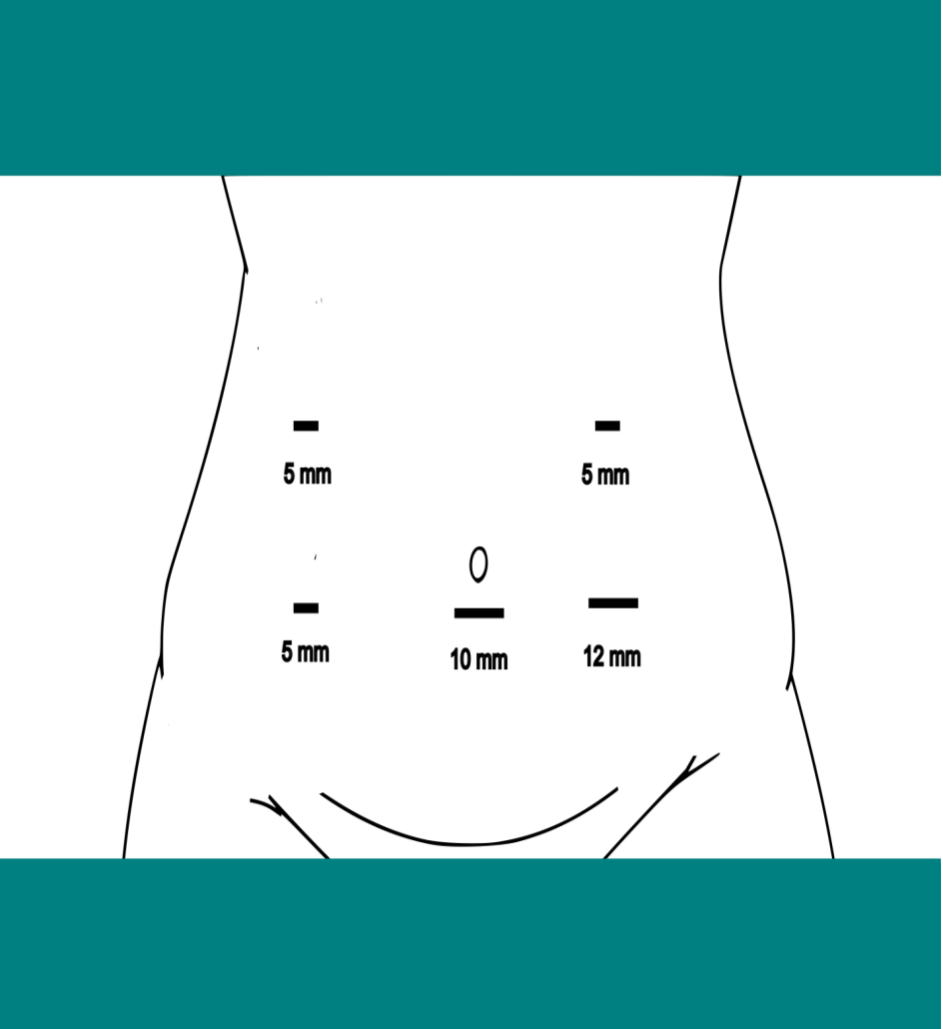
Port placement for laparoscopic resection of right colon cancer with intestinal malrotation.

The patient was positioned supine under general anesthesia, and pneumoperitoneum was established using a Veress needle. A modified port placement strategy was employed to accommodate the abnormal anatomical location of the right colon, with the primary surgeon initially positioned on the patient’s left side (**Figure 3**).

### Initial exploration and malrotation correction

Initial laparoscopic exploration confirmed the absence of metastatic lesions and revealed that the entire colon was located on the left side of the abdomen. Dense adhesions were noted among the ascending, transverse, and descending colon. The right colon was not fixed to the retroperitoneum, and the small intestine was located on the right side. Ladd’s bands were lying in front of the duodenojejunal junction and the duodenum. These findings were consistent with nonrotation IM.

The first critical step was to address the malrotation by carefully dividing Ladd’s bands using a harmonic scalpel. Subsequently, adhesions between the ascending, transverse, and descending colon were separated using a combination of sharp and blunt dissection, preserving the colonic wall integrity and avoiding injury to adjacent structures.

### Mobilization of the transverse colon

Next, the gastrocolic ligament was divided to mobilize the transverse colon, providing access to the retroperitoneal plane. Dissection began cranially at the white line of Toldt, the lateral peritoneal reflection. The ascending colon was mobilized medially, allowing identification of the retroperitoneal space and critical structures such as the ureter and gonadal vessels, which were carefully preserved.

### Identification and dissection of vascular structures

**Figure 4:**
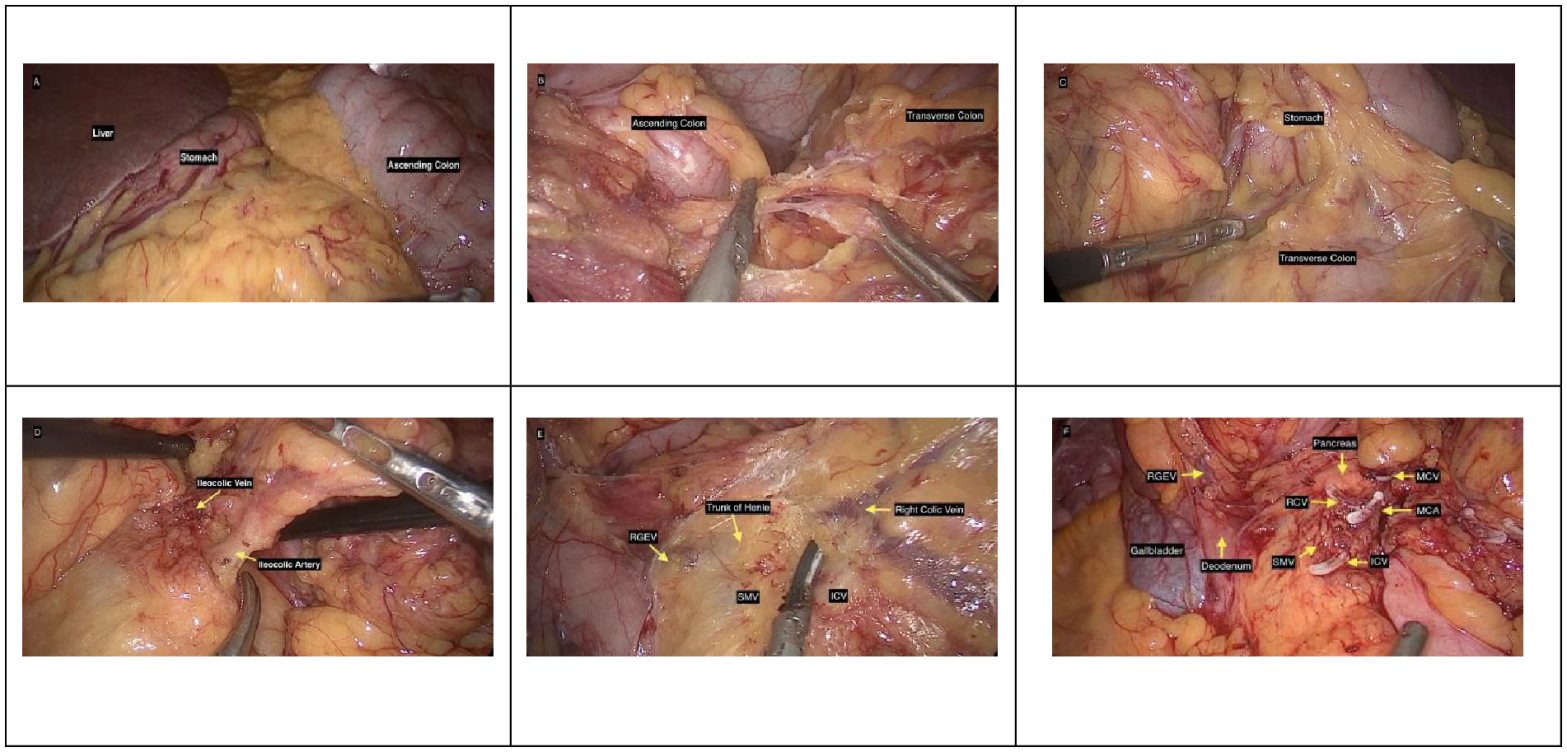
Intraoperative images. **(A)** Image showing the liver, stomach, and ascending colon on the right side. **(B)** Image showing adhesion between the ascending colon and transverse colon. **(C)** Image during dissection of the gastrocolic ligament. **(D)** Image showing the ileocolic vessels. **(E)** Surgical field illustrating vascular anatomy: right gastroepiploic vein (RGEV), trunk of Henle (gastrocolic trunk), right colic vein (RCV), superior mesenteric vein (SMV), and ileocolic vein (ICV). **(F)** Intraoperative photograph of the surgical field after division of vascular structures and organs surrounding it, including the pancreas, RGEV, middle colic vein (MCV), RCV, middle colic artery (MCA), gallbladder, and the duodenum adjacent to the SMV and ICV.

The space between the ascending colonic mesentery and the retroperitoneal tissues was developed. Critical variant vascular anatomy was identified. The SMA and superior mesenteric vein (SMV) were first identified. The ileocolic artery and vein, which originated anomalously from the left side of the SMA, were then identified. The ileocolic artery was dissected up to its origin at the SMA, and the right colic vein was dissected up to the SMV. The middle colic artery was ligated and divided. The Henle trunk and its branches were recognized and preserved. A D2 lymph node dissection was performed to ensure complete removal of all suspicious nodes (**Figure 4**).

### Resection and anastomosis

Once the right colon was fully mobilized, the bowel was exteriorized through a 6 cm midline supraumbilical incision, and an extracorporeal ileotransverse anastomosis was performed using a stapled technique, ensuring adequate resection margins and a tension-free anastomosis. The bowel was then returned to the abdominal cavity, and the incision was closed in layers. The total operative time was 130 minutes, with 50 ml of blood loss.

## Postoperative management and outcome

Pathological examination of the resected specimen confirmed Grade 3 (G3) adenocarcinoma of the ascending colon (pT3N0M0), with clear proximal and distal margins and no evidence of lymphovascular or perineural invasion. The patient’s postoperative course was uneventful, adhering to Enhanced Recovery After Surgery (ERAS) principles with early mobilization and diet advancement (11). She was discharged on postoperative day 9 following the return of bowel function and adequate pain control with oral analgesics. Adjuvant chemotherapy was omitted per multidisciplinary consensus, considering R0 resection, absence of additional high-risk factors (lymphovascular/perineural invasion; R0 resection), and patient comorbidities. Surveillance with serial CEA levels, CA19-9, and abdominopelvic CT scans was carried out as per department protocol. Follow-up examinations at 18 months showed no tumor recurrence.

## Discussion

The laparoscopic management of colorectal cancer in patients with IM demands an in-depth understanding of embryological anomalies to navigate the altered anatomy safely. IM, a rare congenital condition with an adult incidence of 0.0001%–0.19%, originates from disrupted midgut rotation during the 4th to 12th weeks of gestation (2, 12). During this critical period, the midgut normally undergoes a 270-degree counterclockwise rotation around the SMA, followed by fixation to the retroperitoneum (2, 3, 12). Failure of this process results in nonrotation (0°), incomplete rotation (90–180°), or reverse rotation (90°clockwise), leaving the duodenum, cecum, and mesentery unfixed (1–3). Nonrotation IM, as observed in this case, predisposes patients to adhesions and vascular anomalies, including the ileocolic artery originating anomalously from the left of the SMA. These embryological deviations disrupt the typical SMA/SMV relationship, often visualized as SMV inversion (left of SMA) or vertical alignment on imaging, which are pivotal diagnostic clues (2, 5). While IM in adults are frequently asymptomatic and discovered incidentally, symptomatic adults may present with chronic abdominal pain or acute complications like volvulus (4). The coexistence of IM and colorectal cancer represents an exceptional clinical scenario, with fewer than 60 cases documented worldwide—predominantly in Japanese literature. Only 10 cases have undergone complete laparoscopic resection due to aberrant vasculature, visceral displacement, and adhesions compromising oncological dissection feasibility (2, 5). This case of ascending colon cancer with nonrotation IM in a Chinese patient, successfully resected laparoscopically, expands geographic diversity while providing further evidence of minimally invasive techniques’ safety and adaptability in anatomically aberrant scenarios.

Radiological evaluation is essential for diagnosing IM and guiding surgical planning. While Doppler ultrasound can reveal an inversion of the SMA and SMV, with the SMA positioned to the right and the SMV to the left (SMA/SMV inversion) (13). CT remains the gold standard, with sagittal and coronal reconstructions unveiling pathognomonic features such as an “M”-shaped colonic configuration, a left-sided cecum, and the “whirlpool sign” (14). While advanced modalities like 3D-CT angiography (for vascular mapping) (2) and CT colonography (for subclinical IM detection) (15) are described in the literature, conventional CT with multiplanar reconstruction sufficed in this case. It delineated the ileocolic artery’s anomalous origin and dense inter-loop adhesions, enabling preoperative anticipation of dissection challenges. Additional malrotation-specific examinations were omitted as CT provided comprehensive surgical planning data, consistent with current literature (16).

The laparoscopic approach, though technically demanding in IM, adheres to oncological principles while accommodating anatomical complexity. In this patient, adhesiolysis and division of Ladd’s bands were prioritized to approximate normal anatomy. Despite the ileocolic artery’s left-sided origin from the SMA, D2 lymphadenectomy was achieved by tracing the vessel to its root, ensuring oncological rigor. The laparoscopic platform’s magnified view proved invaluable for navigating dense adhesions and vascular anomalies, though procedural success hinged on the surgeon’s familiarity with embryological variations. Modified port placement accommodated the colon’s left-sided malposition, demonstrating the necessity for procedural adaptability. These steps reinforce that adherence to embryological and oncological principles—not the surgical approach itself—dictates outcomes in such anatomically complex scenarios.

This case contributes three key insights to the literature. First, as the first reported Chinese case of IM-associated colon cancer, it broadens the geographic scope of documented cases. Second, it validates the utility of sagittal CT reconstruction in preoperative planning. This approach complements advanced modalities like 3D-CT angiography to optimize surgical precision. Third, it demonstrates that laparoscopic resection in IM-associated colon cancer is feasible with structured anatomical mapping and multidisciplinary collaboration. While the absence of vascular branching anomalies simplified dissection in this case, surgeons must remain vigilant for such variations. This is particularly important in populations where IM is underdiagnosed.

In emergency presentations (e.g., volvulus or bowel ischemia), immediate laparotomy may be mandatory to relieve torsion, assess bowel viability, and perform Ladd’s procedure with extensive adhesiolysis and mesenteric mobilization (4, 17). Resection (e.g., hemicolectomy) is reserved for non-viable bowel or confirmed malignancy. Delayed intervention may risk catastrophic outcomes, including bowel loss and increased mortality (4). However, in select high-volume centers with advanced laparoscopic expertise, Ladd’s procedure may be approached laparoscopically. Contemporary series demonstrate reduced length of stay (LOS) and decreased need for postoperative nasogastric decompression, though conversion rates remain 2-33% (17).

In conclusion, this case underscores that laparoscopic right hemicolectomy with D2 lymphadenectomy can achieve oncological adequacy in IM-associated colorectal cancer when guided by meticulous preoperative imaging and embryological insight. The successful integration of conventional CT with adaptive surgical techniques highlights a pragmatic approach to anatomical complexity, particularly in elderly patients with comorbidities. By fostering global case-sharing and refining standardized protocols, the surgical community can enhance patient outcomes across diverse clinical and resource settings, ensuring that rare anatomical variations like IM no longer preclude the benefits of minimally invasive oncology.

## Data Availability

The datasets presented in this study can be found in online repositories. The names of the repository/repositories and accession number(s) can be found in the article/Supplementary Material.
